# The RCSB protein data bank: integrative view of protein, gene and 3D structural information

**DOI:** 10.1093/nar/gkw1000

**Published:** 2016-10-27

**Authors:** Peter W. Rose, Andreas Prlić, Ali Altunkaya, Chunxiao Bi, Anthony R. Bradley, Cole H. Christie, Luigi Di Costanzo, Jose M. Duarte, Shuchismita Dutta, Zukang Feng, Rachel Kramer Green, David S. Goodsell, Brian Hudson, Tara Kalro, Robert Lowe, Ezra Peisach, Christopher Randle, Alexander S. Rose, Chenghua Shao, Yi-Ping Tao, Yana Valasatava, Maria Voigt, John D. Westbrook, Jesse Woo, Huangwang Yang, Jasmine Y. Young, Christine Zardecki, Helen M. Berman, Stephen K. Burley

**Affiliations:** 1 RCSB Protein Data Bank, San Diego Supercomputer Center, University of California, San Diego, La Jolla, CA 92093, USA; 2 RCSB Protein Data Bank, Department of Chemistry and Chemical Biology, Center for Integrative Proteomics Research, Rutgers, The State University of New Jersey, Piscataway, NJ 08854, USA; 3 Department of Molecular Biology, The Scripps Research Institute, La Jolla, CA 92037, USA; 4 Institute for Quantitative BioMedicine and Rutgers Cancer Institute of New Jersey, Rutgers, The State University of New Jersey, New Brunswick, NJ 08901, USA; 5 Skaggs School of Pharmacy and Pharmaceutical Sciences, University of California, San Diego, La Jolla, CA 92093, USA

## Abstract

The Research Collaboratory for Structural Bioinformatics Protein Data Bank (RCSB PDB, http://rcsb.org), the US data center for the global PDB archive, makes PDB data freely available to all users, from structural biologists to computational biologists and beyond. New tools and resources have been added to the RCSB PDB web portal in support of a ‘Structural View of Biology.’ Recent developments have improved the User experience, including the high-speed NGL Viewer that provides 3D molecular visualization in any web browser, improved support for data file download and enhanced organization of website pages for query, reporting and individual structure exploration. Structure validation information is now visible for all archival entries. PDB data have been integrated with external biological resources, including chromosomal position within the human genome; protein modifications; and metabolic pathways. PDB-101 educational materials have been reorganized into a searchable website and expanded to include new features such as the Geis Digital Archive.

## INTRODUCTION

The RCSB Protein Data Bank (RCSB PDB, http://rcsb.org) builds upon PDB data to enable research and education in structural biology, computational biology and beyond (1,2).

The Protein Data Bank (PDB) is the single global archive for experimentally determined, atomic-level three-dimensional structures of biological macromolecules (proteins, DNA, RNA). The PDB archive is managed by the Worldwide Protein Data Bank organization (wwPDB; http://wwpdb.org) (3), which currently includes three founding regional data centers, located in the US (RCSB Protein Data Bank or RCSB PDB; http://rcsb.org) (1), Japan (Protein Data Bank Japan or PDBj; http://pdbj.org) (4) and Europe (Protein Data Bank in Europe or PDBe; http://pdbe.org) (5), plus a global nuclear magnetic resonance (NMR) specialist data repository BioMagResBank, composed of deposition sites in the US (BMRB; http://www.bmrb.wisc.edu) (6) and Japan (PDBj-BMRB; http://bmrbdep.pdbj.org). Together, these wwPDB partners collect, annotate, validate and disseminate standardized PDB data to the public without limitations on usage.

The RCSB PDB website (rcsb.org) (2,7,8) offers multiple tools for structure query, browsing, analysis and molecular visualization. Users can perform simple searches using the top menu bar search including PDB ID, name, sequence and ligand SMILES; or build complex search combinations of parameters and criteria with the Advanced Search interface. External classification and annotation systems are used to organize PDB data in hierarchical trees for browsing and searching (e.g. Membrane Proteins, Gene Ontology, Enzyme Classification). Visualization options enable exploration of 3D structure, structure/sequence information and correspondences between the two. For example, the Protein Feature View offers a graphic comparison of a PDB sequence with UniProt and other annotations displayed in different tracks.

Here, we describe the latest new features and usability improvements since our last NAR database issue publication (2).

## NEW WEBSITE FEATURES

### Visualization

Structure visualization is fundamental to interrogating 3D macromolecular structures in order to gain an atomic level understanding of biological processes or to explore the mechanism(s) of drug action (9–11). Many stand-alone 3D packages, often with specialized functionality, are available for exploration of 3D structures (12). The need for custom software installation, however, represents a non-trivial barrier to adoption by many students, educators and researchers. For most PDB Users, a simple web-based solution is preferable. Java Applets have served as a mainstay for structure visualization on the web for about two decades. Indeed, Jmol (Jmol: an open-source Java viewer for chemical structures in 3D, http://www.jmol.org/) has been the 3D viewer of choice for the RCSB PDB website and other web resources, numbering in the hundreds. With the advent of recent security precautions, using and deploying such viewers has become cumbersome, and some web browsers (e.g. Chrome) no longer support Java Applets. Oracle recently announced that it would deprecate Java Applets with Java version 9.0.

To provide improved support for 3D graphics, all commonly used web browsers have adopted WebGL as the new technology standard for 3D graphics. In parallel, JavaScript interpreters in web browsers have improved dramatically, and now enable use of graphics applications directly within a web browser. There are a growing number of efforts aimed at developing JavaScript/WebGL based 3D viewers (13). RCSB PDB has supported JSmol (JSmol: an open-source HTML5 viewer for chemical structures in 3D, http://wiki.jmol.org/index.php/JSmol) and PV (14) for some time, and recently added support for NGL.

NGL (15) is a lightweight, highly-scalable 3D viewer that can render large molecular complexes (millions of atoms), without the need for plug-ins, in web browsers on desktops, laptops and smartphones. NGL overcomes important bottlenecks in structure visualization by using: (i) the compact, binary, Macromolecular Transmission Format (MMTF, http://mmtf.rcsb.org) for fast data download and parsing, (ii) a highly optimized data structure and (iii) high speed structure rendering (16).

**Figure 1.**
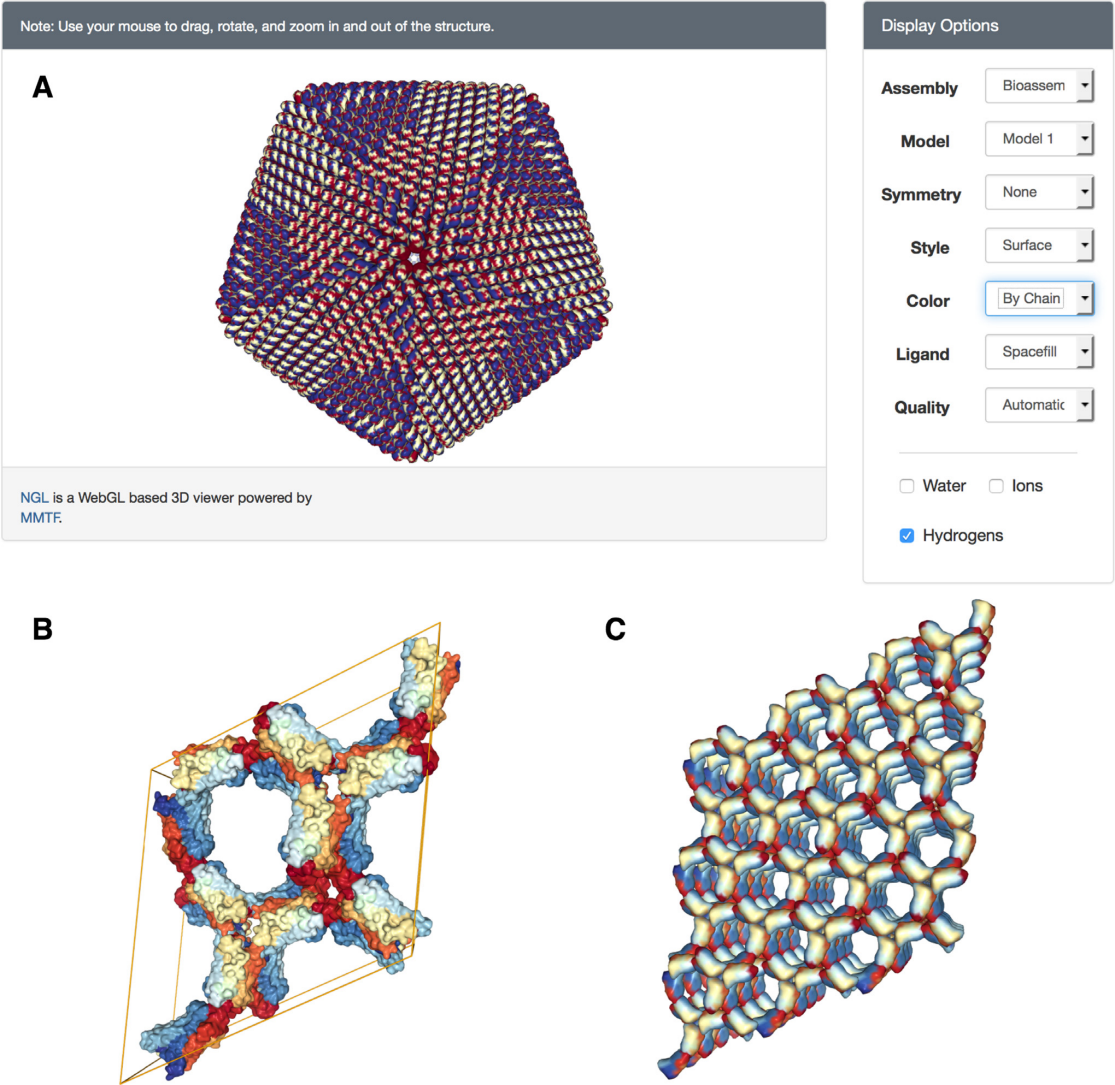
(**A**) NGL viewer and user interface showing a low-resolution surface of the faustovirus major capsid protein (PDB ID 5J7V) generated from a C-alpha representation. This PDB entry consists of 2760 instances of an asymmetric unit, each containing 14 478 atoms. For fast rendering, the surface of the asymmetric unit is created once, and rotation/translation operations are used to create additional copies of the surface. (**B**) Unit cell representation (PDB ID 3PQR) created by applying crystallographic symmetry operations from the asymmetric unit. (**C**) Supercell representation (PDB ID 3PQR) created by translating the unit cell along all three dimensions.

The NGL Viewer interface (Figure 1A) can be launched from the image display box found on each RCSB PDB *Structure Summary* page. Due to its ease in handling very large structures, NGL Viewer is now the only tool supported by the RCSB PDB for archival entries with >10 000 residues in either the asymmetric unit or the biological assembly. For such entries, a backbone-only representation of the biopolymer chains is displayed by default to reduce the file download and parsing time. Display style and quality are optimized so that the viewer will run efficiently on both mobile and desktop systems, but can be customized for more powerful systems or for generation of high-resolution images.

### Integration with biological resources

RCSB PDB resources provide a ‘*Structural View of Biology*’ enabling a deeper understanding of important biological processes and mechanisms in context with 3D structural information from the PDB archive. To accomplish this end, the RCSB PDB website integrates PDB data with a large number of open access primary and derived data resources developed and provided by the community. External data can be accessed from individual *Structure Summary* pages, and in many cases, can be used in generating searches and preparing tabular reports. Supplementary Table S1 provides a summary of these external resources.

Newly integrated data enable improved analysis of chromosomal position in the human genome and PDB structure; protein modifications; and metabolic pathways.

### Mapping genomic position to 3D structure

**Figure 2.**
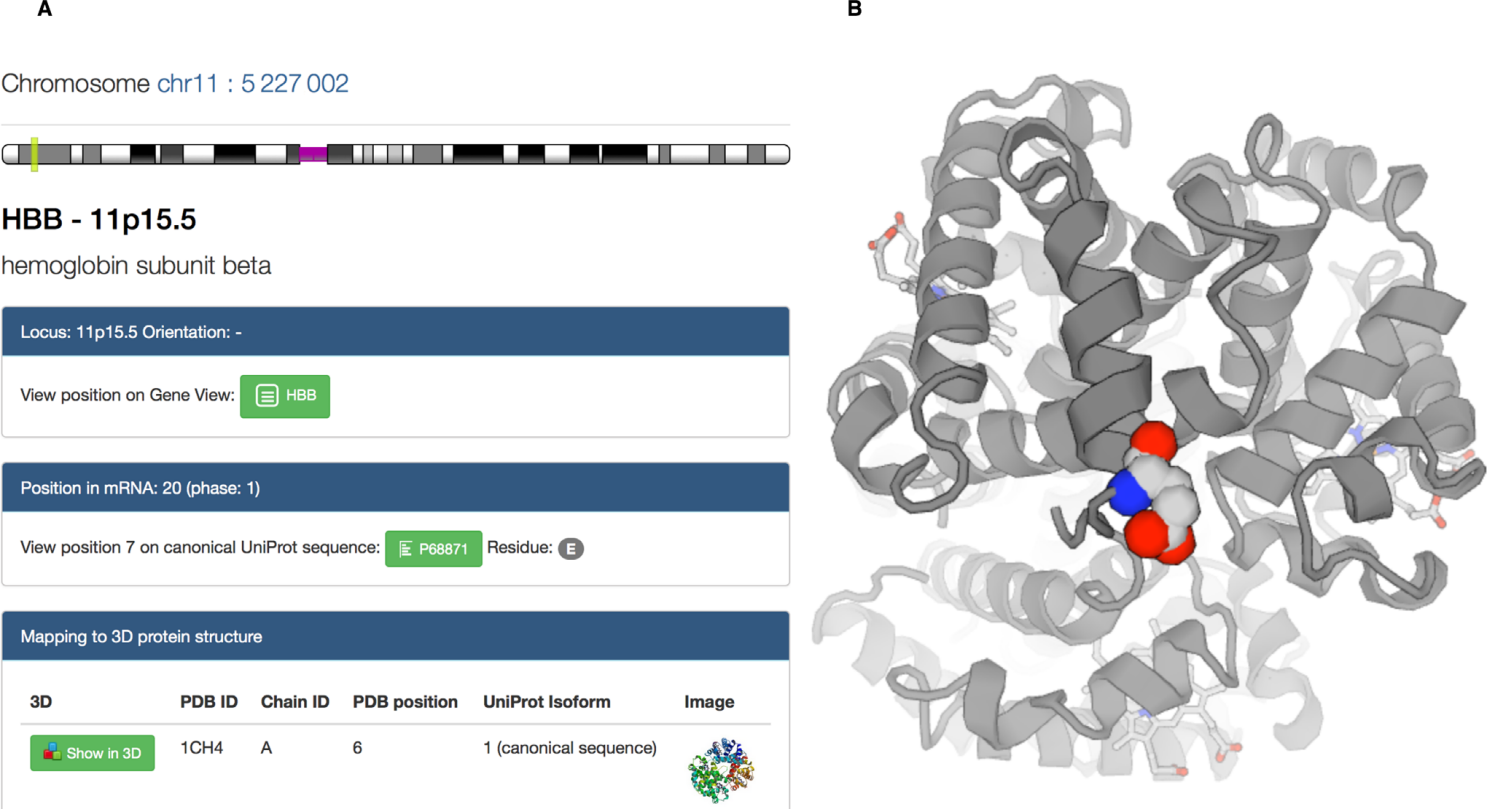
(**A**) Mapping tool from human genomic coordinates to UniProt Isoforms and corresponding locations in a PDB structure. The selected position corresponds to the site of a genetic variation in the hemoglobin beta chain that is known to cause sickle-cell disease. (**B**) The corresponding position on the protein can be visualized in 3D (PDB ID 1CH4). A similar 3D view is available from the *Protein Feature View*.

To facilitate the integration with human genomic information, a new analysis tool (Figure 2) maps any chromosomal position in the human genome to PDB data by matching alternative transcripts that might be available for a gene to the corresponding UniProt isoforms (17).

### Protein modifications

Proteins are chemically modified at different stages during protein synthesis, including pre-translational (i.e. N-formylation), co-translational and post-translational events. Such modifications can modulate or expand the repertoire of protein function. They may also be involved in disease processes. We have mapped Protein Modifications (PMs) in the PDB to the RESID database of PMs (18,19) and the associated Protein Modifications Ontology PSI-MOD (20). PM definitions and the software to identify them are available from the BioJava project (21) (https://github.com/biojava/biojava/tree/master/biojava-modfinder).

PMs can be explored by using the *Browse by Annotations* feature accessible at the top of the RCSB PDB homepage. PMs can be browsed by two PSI-MOD Ontologies: (i) categorized by either the amino acid involved in the modification or (ii) by the chemical process involved in the formation. PMs can also be searched using the *Advanced Search* by name, keyword, RESID ID, PSI-MOD ID and the wwPDB Chemical Component Dictionary ID.

For PDB entries containing PMs, the *Structure Summary* page offers several viewing options. The *Annotation Tab* lists the PMs within a structure. The *Sequence Tab* visually maps protein modifications on a sequence diagram, and the *3D View Tab*, contains a list of PMs that can be mapped onto the 3D structure using JSmol.

### Metabolic pathways

Metabolic pathways are sequences of chemical reactions that occur within a cell. Enzymes act on substrates, also known as metabolites, to perform a series of chemical reactions, with the product of one chemical reaction becoming the substrate for the next enzymatically catalyzed reaction in the pathway. Metabolic pathways typically involve important processes enabling cells to utilize energy or perform biosynthesis of vitamins and other co-factors, and many other essential mechanisms required for cell viability.

**Figure 3.**
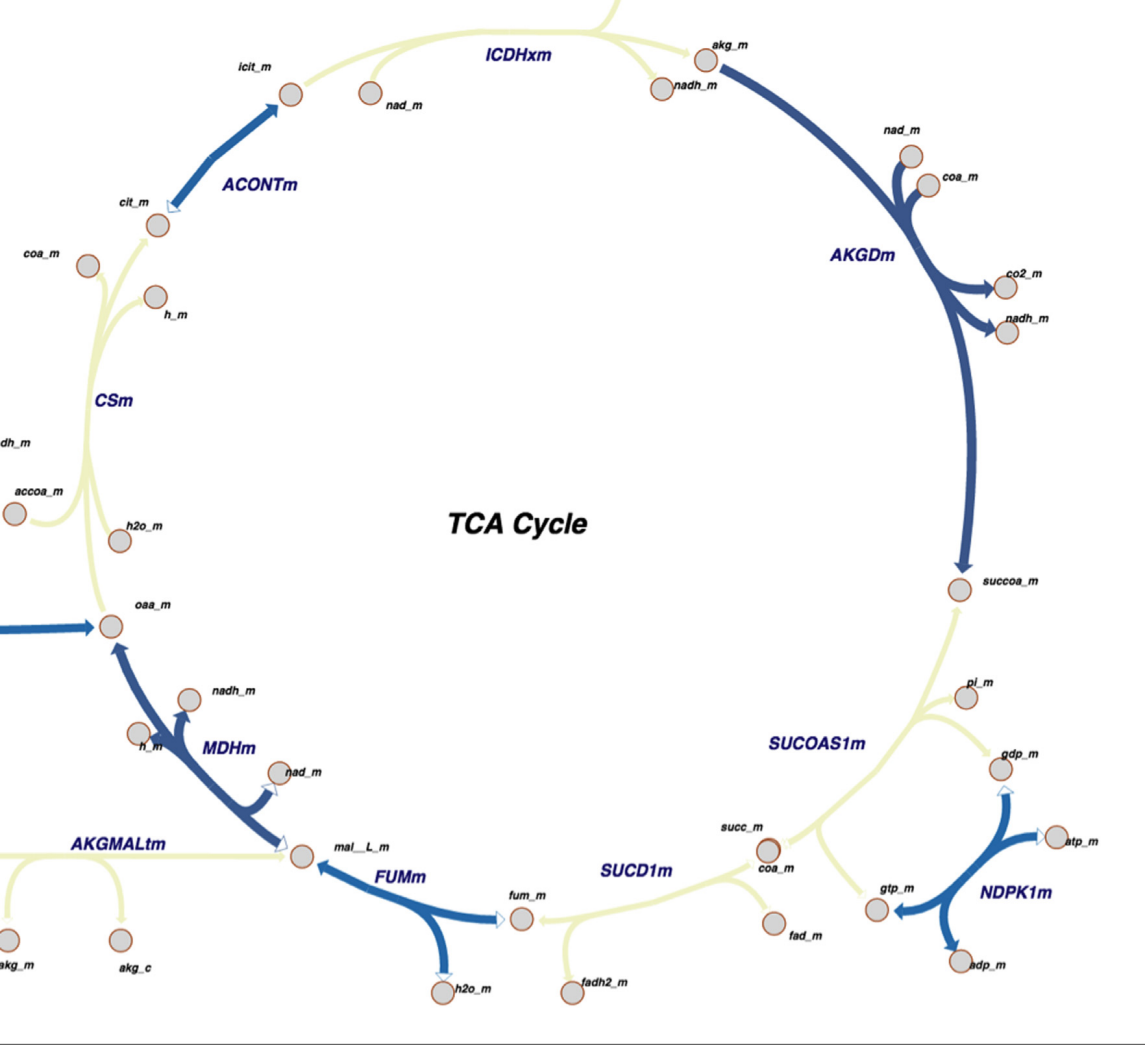
The new *Pathway View* provides graphical representations of metabolic pathways using Escher. Shown here is the Tricarboxylic acid (TCA) or Krebs cycle for human. Color-coding indicates parts of the pathway for which 3D structures are available in the PDB (blue: PDB structures available; yellow: structure can be inferred *via* homology modeling).

An increasing number of 3D structures of proteins responsible for catalyzing reactions within metabolic pathways are being submitted to the PDB. To accommodate a structural view of biological chemistry, we offer a new way to explore metabolic pathways that maps reactions and metabolites to the atomic-level data available in the PDB. The first large-scale structural mapping of metabolic networks in an organism was for *Thermotoga maritima*, described by Zhang *et al.* (22). Our new pathway view (Figure 3) supports browsing through well-characterized metabolic pathways and visualizing 3D structures of enzymes and metabolites comprising such pathways with the Escher visualization tool (23).

Mapping of enzymatic reactions to three-dimensional structures is based on data provided by the GEM-PRO project (24). Reconstructions of pathways are based on the BiGG Models (25), a knowledgebase of biochemically, genetically and genomically structured genome-scale metabolic network reconstructions. A genome-scale network reconstruction represents a curated knowledge-base comprising many different data types and sources, including high-quality genome annotation, assessment of biochemical properties of gene products and a wide array of physiological functional information (25). Over the past three years, a deluge of ‘omics’ data have enhanced opportunities to characterize metabolites, reactions, proteins and genes in an organism-specific manner. There are now more than 75 high-quality, manually-curated genome-scale metabolic models for organisms ranging from bacteria to humans (25). At the time of submission of this manuscript it is possible to visualize selected pathways for human and *E. coli* at the RCSB PDB website. We are collaborating with the systems biology community to increase the number of supported pathways and model organisms in this view. Genome scale models are mapped to UniProt identifiers. To provide the most up-to-date link from pathway to PDB, we update the underlying data on a weekly basis using the UniProt to PDB mappings are available from the SIFTS initiative (26).

### Searching and reporting

**Figure 4.**
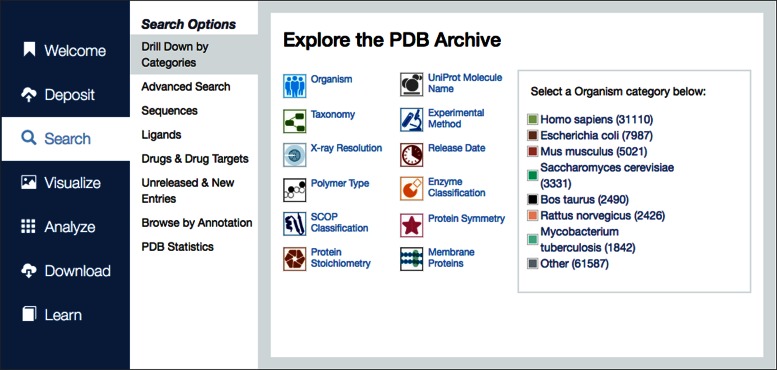
The left-hand menu on the homepage provides easy navigation to high-level tasks. In this example *Search* is selected. The middle panel lists available search options, and in this example, *Drill Down by Categories* is selected. The associated right panel exposes further drill-down options to select a specific subset of PDB entries.

RCSB PDB aims to continuously improve the User experience on desktop, laptop and mobile devices. While the top text search bar on the rcsb.org website is the main entry point for many Users, we redesigned the homepage to expose the rich collection of website features grouped into functional categories to offer a ‘one-stop-shopping’ experience. Specifically, the left-hand menu and top menu bar provide access to all high-level tasks, from depositing a structure, to searching, visualizing and analyzing PDB data (Figure 4). Finally, the download section includes functionality for file export and describes available RESTful web services. The *Learn* section guides the User to the PDB-101 educational portal of the RCSB PDB. The homepage also highlights the latest Molecule of the Month, new PDB entries, new features and weekly news items. This page has been optimized for viewing on mobile devices.

**Figure 5.**
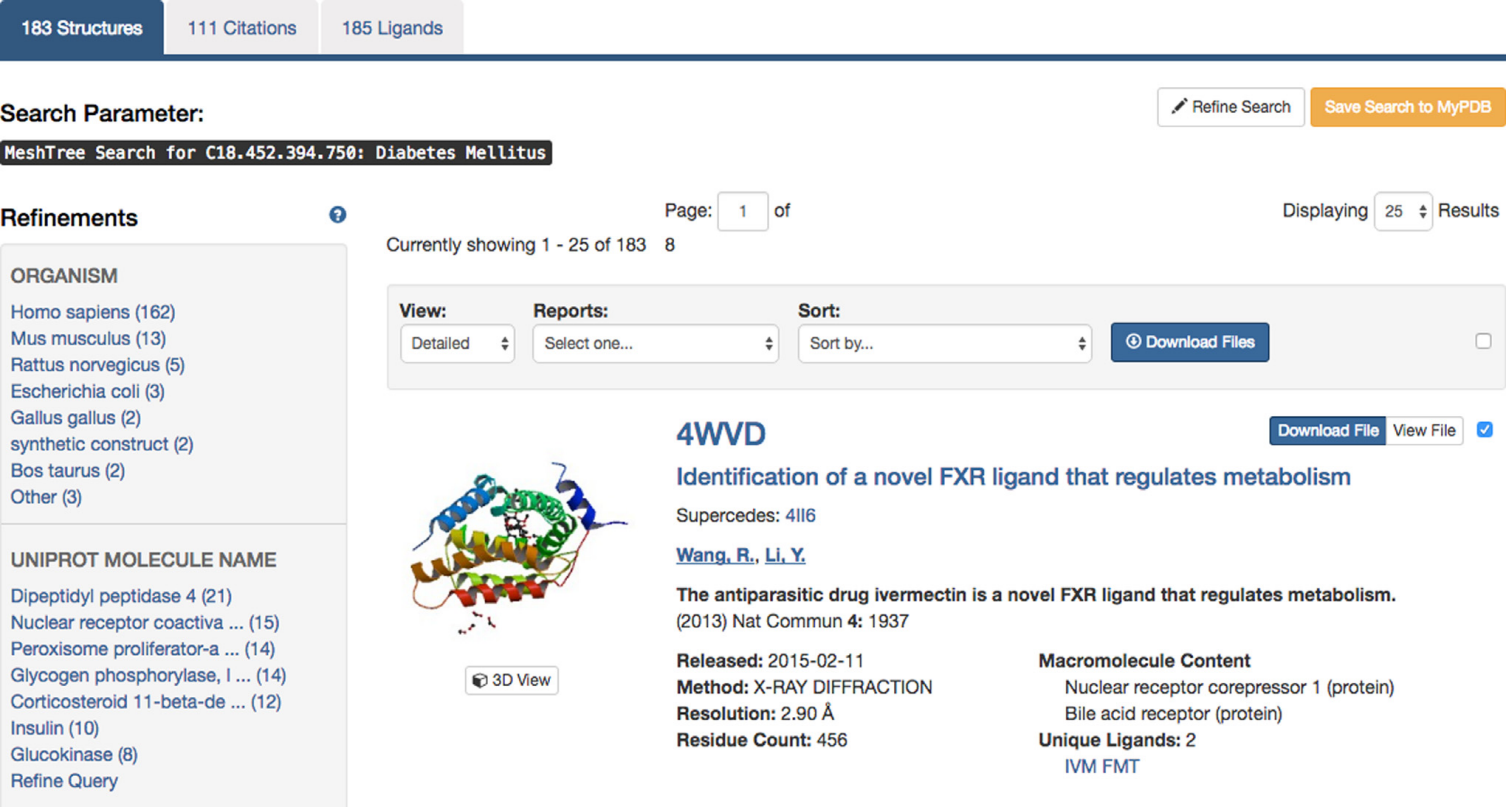
Top section of the *Search Results* page after a MeSH term search for *Diabetes Mellitus*. Only a single hit is shown here for brevity. The search results can be refined by clicking on any of the terms in the left-hand panel, e.g. *Homo sapiens* to limit the result set to structures with protein chains derived from human.

Recently, we improved the look and feel of search result display options. A faceted browsing (drill-down) panel on the left enables users to quickly narrow down the search results, and a large, simple right-hand panel with the search results, supports navigation to browse through multiple results pages. Search outcomes are subdivided using several tabs (Figure 5): (i) structure hits, (ii) unique set of primary citations and (iii) unique set of ligands present in the structure results. The *View* option exposes alternative representations of the search results, including a *Condensed* version for quick browsing, a *Gallery* view for visual browsing and a *Timeline* view for browsing in chronological order. The *Reports* options allows for User export of search results in tabular form suitable for spreadsheet applications and data analysis software. Search results can also be sorted by PDB ID, release date, residue count and resolution limit. Atomic coordinates, polymer sequences or ligands, can be downloaded using the *Download File(s)* buttons. The query can be saved in a User *MyPDB* account for future use, or to set up automatic email notifications if a newly released structure matches the stored query.

#### Wild type search

A new *Advanced Search* option for wild-type protein selects protein sequences that do not contain mutations, as judged by comparison with the reference sequence archived by UniProt (27). Some PDB entries include expression tags, point mutations or deletions/insertions added by the Depositor. Such entries can be excluded from the search using this mechanism. PDB entries frequently contain only a portion of the referenced UniProt sequence. The *Percent coverage of UniProt sequence* option defines how much of a UniProt sequence needs to be contained in the PDB entry to be included in a *bona fide* search hit.

#### Structure summary page

**Figure 6.**
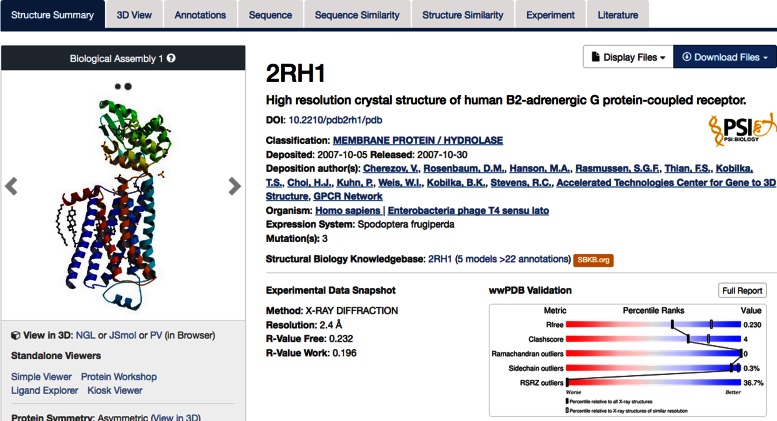
Upper portion of the *Structure Summary* Page for PDB ID 2RH1. Tabs are used to navigate to details including annotations, sequence level information, sequence and structure similarity, experimental details and literature associated with an entry. Blue *query by example* links launch queries for the selected term. The wwPDB Validation Report slider provides a high level view of various geometric and model fit metrics.

After searching, the next step in a typical User workflow involves viewing the *Structure Summary* pages of one or more PDB entries (Figure 6). *Structure Summary* pages are themselves organized with various tabs:

- Structure Summary: High level overview
- 3D View: Interactive web-based molecular viewers
- Annotations: Protein domain and functional annotations
- Sequence: Diagram of polymer sequences, secondary structure, binding sites, protein modification sites
- Sequence Similarity: Clusters of similar protein sequences
- Structure Similarity: Structural neighbors of protein domains
- Experiment: Structure determination details
- Literature: List of publications referencing the PDB ID

The *Literature* tab, if available, contains a link to papers in PubMed that cite the primary citation. In addition, PDB data mentions (28) appearing within the full text of PubMedCentral Open Access journals (http://www.ncbi.nlm.nih.gov/pmc/tools/openftlist/).

**Figure 7.**
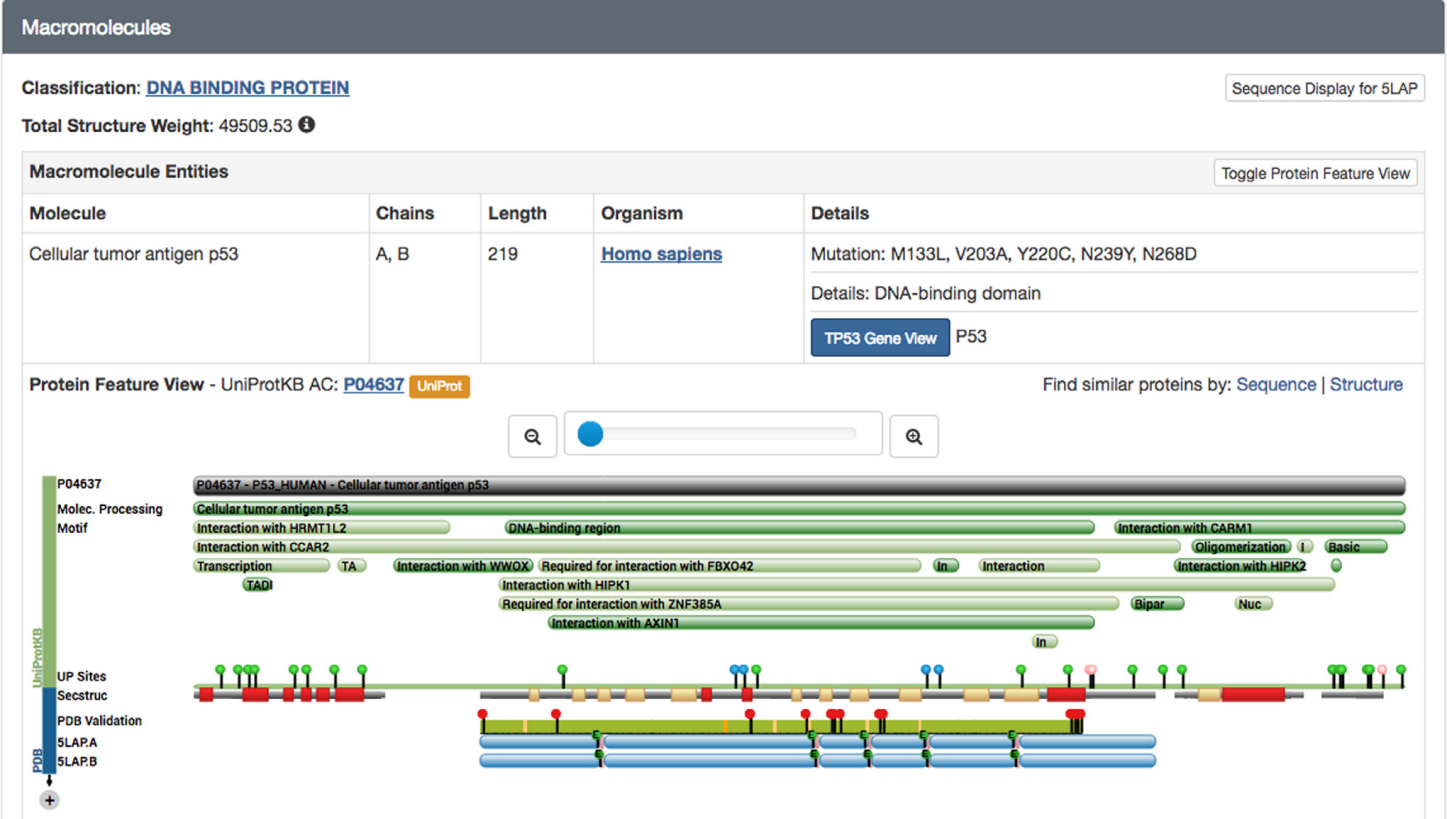
Macromolecules section of the *Structure Summary* Page for PDB ID 5LAP. Top portions summarize detail regarding the polymer chain. A link is provided to the UniProt sequence, and two links allow the user to find similar protein chains by either sequence or structure similarity. The bottom portion shows a simplified version of the *Protein Feature View*, which includes a PDB Validation track.

The upper portion of the *Structure Summary* page shows a molecular image and links to 3D viewers, and a short summary including Deposition title, Depositor names, Deposition and Release dates, Source organism and brief details concerning the method of structure determination. The wwPDB Validation Report slider provides a graphical summary of structure quality (see below for discussion of Structure Validation). Next, the macromolecular polymers present in the entry are described (Figure 7). Each unique polymer entity is listed in the Macromolecules section, with polymer name, chain identifiers, chain length and details, such as mutations, if any. A link to the *Gene View* is provided to map this structure into a genomic context (17). If the polymer sequence can be mapped to a UniProt sequence, a simplified Protein Feature View diagram (17) is also included.

**Figure 8.**
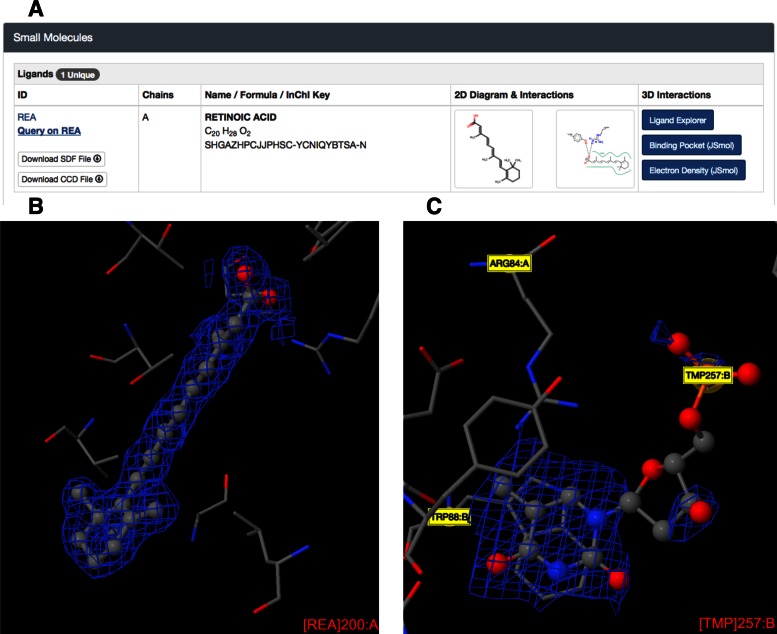
(**A**) Small molecule section of the *Structure Summary* page (PDB ID 1CBS) with 2D chemical diagram, 2D interaction diagram (PoseView) and links to 3D viewers, including an electron density view around the ligand. (**B**) SigmaA-weighted 2m|Fo|-D|Fc| electron density map for a retinoic acid ligand (REA in PDB ID 1CBS) contoured at 1 sigma level, showing excellent correspondence with the atomic model. (**C**) SigmaA-weighted 2m|Fo|-D|Fc| electron density map for a thymidine-5′-phosphate ligand (TMP in PDB ID 3HW4) contoured at 1 sigma level, showing poor correspondence with the atomic model.

The Structure Summary Page also includes a Small Molecules (Figure 8A), Experimental Data & Validation and Entry History section.

### Structure validation

In consultation with community experts, the wwPDB has assembled a series of experimental method-specific validation task forces (VTFs) to develop recommendations for structure quality assessment. Recommendations from the wwPDB X-ray VTF (29) have been implemented and the new wwPDB Validation Reports have been available since early 2014. In March 2016, these reports were updated with the latest archive statistics, obtained from the 2015 snapshot of the PDB. As of May 2016, wwPDB Validation Reports have been made available for all entries, incorporating the recommendations of the NMR (30) and 3D cryo electron microscopy (31) VTFs. Validation reports for all three experimental methods use a common system for analyzing the quality of polymer atomic models (including deviations from standard geometry, close contacts and polymer linkage issues). For X-ray structures, various measures of agreement with experimental structure factor data are provided. For NMR structures, measures of agreement with NMR restraint and chemical shift data are reported.

wwPDB Validation Reports can be generated before and during structure deposition to help identify and resolve potential issues prior to finalizing a new PDB entry as part of the wwPDB OneDep system (http://deposit.wwpdb.org/deposition/) for structure deposition, annotation and validation (Jasmine Y. Young *et al.*, submitted). A summary description of these reports is available (http://www.wwpdb.org/validation/validation-reports). Validation data are available both in machine-readable files (XML) and as human readable validation reports (PDF format).

The RCSB PDB presents both the official wwPDB Validation Report and the validation sliders are available on the *Structure Summary* page (Figure 6). In addition, a PDB Validation track has been added to the Protein Feature View (Figure 7) for the specific PDB entry. This track is color coded by the number of geometry outliers present in each residue in the polymer chain (green: no outliers; yellow: 1 outlier; orange: 2 outliers; and red: 3 or more outliers). In addition, residues with poor fit to the electron density map (Real Space R-factor Z-score or RSRZ > 2) are denoted with red tags. (N.B.: RSRZ values are computed relative to other structures in the archive obtained at similar diffraction data resolution.)

To help the User assess the goodness of fit between the atomic model of the ligand and the data coming from the crystallography experiment, the RCSB PDB website displays the electron density map in the vicinity of each ligand (Figure 8; accessible from the Small Molecule section of the *Structure Summary* page). These electron density maps (sigmaA-weighted 2m|Fo|-D|Fc|) are calculated with both the observed crystallographic data (|Fo| or observed structure factor amplitudes) and the atomic model of the structure using the method of Read (32). These calculations include correction factors for errors in both the measured experimental data and the atomic model of the structure.

Where possible, we provide links *via* digital object identifiers (DOIs) to the primary X-ray diffraction data images used to generate experimental data archived in the PDB (i.e. |Fobserved| or observed structure factors), which were in turn used to generate the archived atomic coordinates. To date, we have established automated linking procedures for the Integrated Resource for Reproducibility in Macromolecular Crystallography (http://proteindiffraction.org/), the Structural Biology Data Grid (https://data.sbgrid.org/) (33) and the Store.Synchrotron Data Store hosted by the Australian Synchrotron (https://store.synchrotron.org.au/) (34). Additional linkages will be established with other primary diffraction data image repositories. Using the *Advanced Search* for ‘External Links’, all structures for which diffraction data images are available can be retrieved. Currently, about 3000 such data sets have been linked to PDB entries. When available, links are displayed in the *Experimental Data & Validation* section of the *Structure Summary* page.

## NEW DATA SERVICES

### Data file downloads

The download tool, available from the *Download* panel on the home page, supports batch download of multiple structure, experimental data, sequence and ligand files in different file formats, with and without compression. In addition to FTP file access, data files are now accessible through both HTTP and secure HTTPS protocols (http://www.rcsb.org/pdb/static.do?p=download/http/index.html) to simplify access by other web resources. This functionality provides access to PDB atomic coordinates (.pdb, .cif, .xml), experimental data (structure factors for X-ray, restraints and chemical shifts for NMR) and small molecule data files [Chemical Components (35) and Biologically Interesting Molecules (36,37)].

### Large structure support

The wwPDB has made changes in how large structures (such as ribosomes) were distributed in the archive. Historically, entries with >62 chains and/or 99 999 ATOM lines were ‘split’ into multiple entries due to the size limitations of the PDB File Format. In 2014, the wwPDB began to distribute large structures as single entries represented in the archive in both PDBx/mmCIF and PDBML formats, and as a tar file containing a collection of ‘best-effort,’ limited PDB-format files with authorship, citation details and coordinate data and a mapping file that contains the mapping between the chains present in the large entry and the chains present in the limited PDB-format files.

RCSB PDB *Structure Summary* pages provide access to display and download these data files. Users searching for ID codes of historically ‘split’ entries are automatically redirected to the combined entry.

A separate directory in the PDB FTP archive contains a TAR file including a collection of ‘best-effort’, minimal, PDB format files for large Structures that contain authorship, citation details and atomic coordinate data, and an index file that contains the mapping between the chains present in the large entry and the chains present in the limited PDB-format files. DOIs for large Structures point to these TAR files.

Large Structures are exclusively distributed in the main PDB FTP directory in PDBx/mmCIF and PDBML formats, including biological assembly files. Structures that do not exceed the limitations of the PDB format will continue to be provided in both the PDBx/mmCIF and PDBML formats, as well as the legacy format PDB files in the archive for the foreseeable future.

### Data export using tabular reports

The tabular report functionality, available from the *Reports* menu on the Search Results Page (Figure 5), was historically limited to per structure and to per entity (unique polymer chain) reports. We have expanded this functionality to allow preparation of reports on a per polymer chain level (i.e. 4HHB.A, represents chain A in PDB entry 4HHB). By using the *Advanced Search*, Users can formulate a query for a list of chain IDs and then create and save a chain-based tabular report. In addition, sequence cluster information from 30–100% amino acid sequence identity, recommended and alternative UniProt names, gene names and synonyms assigned by PDB biocurators, have been made available for tabulation.

## NEW EDUCATIONAL SERVICES

PDB-101 is an online portal for teachers, students and the general public to promote exploration in the world of proteins and nucleic acids (38). The website has been recently redesigned and is available at http://pdb101.rcsb.org. New features include keyword and text searching to improve access to more than 200 Molecule of the Month articles (39), paper models, posters, animations, curricular modules and other materials developed by the RCSB PDB to support exploration and extended learning.

The reorganized Browse feature lists PDB-101 materials by topics (e.g. the immune system and renewable energy). A Guide to Understanding PDB Data offers an introduction to more PDB-specific information: PDB Data, Visualizing Structures, Reading Coordinate Files and Potential Challenges (including an explanation of the difference between the biological assembly and asymmetric unit of a model coordinate entry). Recently developed curricular modules and information about competitions/challenges for students, organized by or in collaboration with RCSB PDB are also available here.

A new highlight of the redesigned PDB-101 resource is the Geis Digital Archive. Irving Geis (1908–1997) was a gifted artist and long-term friend of the RCSB PDB. Geis pioneered molecular visualization for structural biologists by drawing and painting more than 700 iconic images. In collaboration with the Howard Hughes Medical Institute, the RCSB PDB is publishing many of his illustrations in the context of their molecular structures from the PDB archive. Images are available for download for non-commercial use for each illustration shown (http://pdb101.rcsb.org/geis-archive/gallery-by-name).

## SUMMARY

The PDB archive is growing at a yearly rate of ∼10%, and structural biologists are determining atomic level 3D structures for a growing number of ever larger and more complex molecular assemblies. This growth has led to a number of challenges for interactive visualization, providing a biologically relevant context to 3D structures, data distribution and structure quality assessment. In this paper we have highlighted recent improvements in ‘Data Out’ functionality undertaken by the RCSB PDB team (1) to provide Users with a ‘*Structural View of Biology*’. Improvements are reported for 3D visualization of large complexes using NGL Viewer and the compact Macromolecular Transmission Format (MMTF); integration of 3D structural information with additional publicly available data resources; mapping of genomic positions to protein sequence positions and locations in 3D structure to provide a platform to analyze the effect of genomic mutations on protein structure; and mapping of 3D protein structures into metabolic pathways that explain relationships between enzymes and metabolites. Improvements to the *Home* Page, *Search* Results and *Structure Summary* page have enhanced the User experience on desktop, laptop and mobile devices. Display of new wwPDB Validation Reports and graphical validation sliders help our Users assess the quality of each structure archived in the PDB. New download options now support large Structure download. The RCSB PDB educational section, PDB-101, has been reorganized to simplify navigation and add search capabilities. Within PDB-101, the Geis Digital Archive offers a unique perspective on the early days of protein structure determination, when 3D computer graphics was in its infancy. The RCSB PDB website continues to be further improved and integrated with other biological data resources to meet current and future requirements of biomedical researchers, educators and their students and specialists in structural biology or structural bioinformatics.
